# Irritable Bowel Syndrome: Patient-Provider Interaction and Patient Education

**DOI:** 10.3390/jcm7010003

**Published:** 2018-01-02

**Authors:** Albena Halpert

**Affiliations:** 1 Boston University School of Medicine, Boston, MA 02118, USA; ahalpert@pmaonline.com; Tel.: +1-978-557-8921; 2 Pentucket Medical Associates, Haverhill, MA 01843, USA

**Keywords:** Irritable Bowel Syndrome (IBS), patient education, patient physician relationship, patient provider relationship, patient provider interaction

## Abstract

The Patient-Provider (P-P) relationship is the foundation of medical practice. The quality of this relationship is essential, particularly for the management of chronic illness such as Irritable Bowel Syndrome (IBS), since it correlates with disease improvement. A significant aspect of fostering the P-P relationship is providing effective patient-centered education about IBS. An effective education empowers the patients to achieve the main therapeutic goals: to reduce symptoms and improve quality of life. Method: A literature search of PubMed was conducted using the terms “Irritable Bowel syndrome”, “Patient Physician Relationship”, “Patient Provider Relationship”, and “Patient Physician interaction”. Preference was given to articles with a clearly defined methodology and those with control groups if applicable/appropriate. This article provides a review of the literature on Patient-Provider interaction and patient education as it relates to IBS and provides practical recommendations on how to optimize this important relationship.

Throughout the history of medicine, clinicians have recognized that their relationship with the patient can be as healing as the treatment that they recommend. In modern terms, the Patient-Provider (P-P) relationship has a therapeutic effect, comparable or sometimes greater than that of pharmacological agents. The effects of the P-P interaction on placebo (inert substance that provokes perceived benefits) or nocebo (inert substance that causes perceived harm) provide strong evidence that the context of the P-P encounter impacts clinical outcomes. If the effects of prescribing a medication may be enhanced by a friendly and empathic approach or reduced by overly focusing on side effects, then the impact of the P-P interaction should be as highly appreciated in clinical practice as we appreciate the effects and side effects of pharmacological agents [1].

Studies using brain imaging have challenged the notion that placebo effects primarily reflect a response bias. The therapeutic context induces biochemical pathways in the patient’s brain that may enhance or reduce visceral pain or modify the effects of interventions/treatments [2,3]. In clinical practice, placebo and nocebo are psychobiological phenomena, based on expectancy and conditioning, and cannot be separated from clinical practice. These phenomena are very common and clinically significant, yet under-recognized. They are also inseparable from the disease course and the treatment response, thereby creating a challenge for pharmaceutical trials [4]. Therefore, as medicine is moving towards utilizing biomarkers and genetics to create more targeted and more personalized treatments, the P-P communication will equally need to be studied and personalized [5]. Unfortunately, aside from mental health professionals, most health care providers today rarely view their relationship with the patient as a therapeutic tool. This relative “neglect” of the importance of the P-P relationship in medical care is rooted in the western medical tradition and training, which favor morphology and treatment of disease over treating the illness (the disease experience). Furthermore, the P-P relationship is increasingly being challenged by a variety of factors such as the lack of emphasis on communication skills training, the reimbursement structure favoring procedural aspects of medicine, time pressures to see more patients in shorter visits, the influence of internet and direct pharmaceutical advertising, government regulations, and the rising costs of medical care and medical litigation. The weakening of the P-P bond is unfortunate since an effective P-P relationship has multiple benefits for both patients and providers: patients benefit from an improved diagnosis, increased satisfaction, greater adherence to medications, and improved self-management. Providers experience improved practice efficiency, reduced medical costs, a decreased risk of malpractice claims, and increased job satisfaction [6,7,8,9,10,11,12]. According to a recent national survey of American physicians, the most satisfying aspect of medical practice is “patient relationships”, as identified by 71% of participants, followed by intellectual stimulation, while the least satisfying aspects were regulations and the lack of clinical autonomy [13]. 

When managing Irritable Bowel Syndrome (IBS), the importance of therapeutic P-P interaction is possibly more important than in other better defined chronic illnesses. Poorly understood pathophysiology, the absence of biological markers or curative treatments, and psychiatric co-morbidities in patients with severe IBS, make this condition “less legitimate” and contribute to a vicious cycle of unnecessary testing, vague diagnosis, repeat visits, and ultimately poor health outcomes. Patients often feel embarrassed to discuss bowel symptoms, and feel misunderstood, isolated, and even distrusted by their family members and health care providers. At the same time, health care providers may feel frustrated by a lack of definitive treatments, underestimate the impact of IBS on patients’ lives, and have negative feelings towards IBS patients—thus undermining the therapeutic relationships.

## 1. The Patients’ Perspective of IBS 

Three major themes seem to determine IBS patients’ illness experience: (1) feeling of frustration from lack of control; (2) a sense of isolation; and (3) dissatisfaction with the available treatments, information received, and the health care system in general [14,15].

### 1.1. Frustration 

Patients with IBS frequently voice frustration over the inability to anticipate, prevent, or control the symptoms of IBS. Patients’ comments include: “*I have no control over my life. I never know if a specific food or situation will trigger an episode. There is no rhyme or reason when it comes to my IBS.*” Many patients report that they need to know where the bathrooms are located at all times and would rather miss social events, travel, or work opportunities than risk the embarrassment and stress of having unexpected symptoms. They feel a lack of control over their symptoms; “IBS is controlling my life”. Added to the perceived lack of control is the feeling of not being understood or validated by family members, co-workers, friends, and the medical profession. It is common for patients to report, “*No one understands how frustrating and embarrassing it is to have bowel symptoms at the worst possible times. No one understands it is a real condition and not in my head.*” The unpredictability and taboos of IBS often make the patients suffer, feel constrained and dependent, and struggle to preserve dignity [16]. Clinically important is that this frustration relates to feeling helpless, which in itself is linked to an inability to cope, poor self-image, increased disease severity, and negative impact on health outcomes [17,18].

### 1.2. Social Isolation 

Social isolation is a major theme in the patients’ IBS experience. Due to the nature of the IBS symptoms related to bowel function, the social isolation associated with IBS is arguably greater than for other chronic conditions. Discussion about bowel habits is somewhat taboo, or at the least socially unacceptable, and therefore embarrassing. Patients may choose to suffer alone without sharing their experiences with family, friends, or sometimes even with their healthcare providers. About 50% of IBS patients do not inform their family members and friends about being diagnosed with the disease [19]. Concerns about bowel habits, feeling “unclean”, and interference with dating, intimacy, and sexuality are very common among patients with IBS. Patients frequently report a loss of freedom, shame, and embarrassment. Importantly, these thoughts could lead to behaviors that encourage further isolation, such as avoidance, withdrawal, poor self-esteem, and missing work, leisure, and social events. Moreover, the feelings of being misunderstood or judged regarding the legitimacy of the emotions related to these experiences, and the behaviors in dealing with it, may further increase the sense of isolation. Confounding this isolation on a societal level is the relative lack of public awareness or discussion about IBS when compared to other chronic illnesses such as asthma or diabetes [20,21]. Social isolation could be a barrier to daily function and to connection with others who could provide support (e.g., other patients or support groups that may be very helpful).

### 1.3. Dissatisfaction with the Medical System and Need for More Information 

Only a small proportion of people who suffer from IBS seek medical care for it. Patients with moderate to severe disease severity who visit their providers have high health care utilization and frequently report being disappointed with their medical care and persistent symptoms. According to a national online survey, less than one-third of study participants were satisfied with the medications they used for IBS. Two out of five IBS patients stated that they are “not at all satisfied” with their care. Furthermore, 14% of the study participants reported that they would accept a risk of 1/1000 chance of death for a treatment that would free them from IBS symptoms [22]. Healthcare professionals were generally viewed as not sufficiently supportive and not providing enough information or guidance related to the management of IBS symptoms [23,24]. Overall, patients tended to feel they were not taken seriously, labeled as “neurotic”, stigmatized, and let down by their health care providers. Furthermore, patients with severe IBS described that they were not always acknowledged as a person and felt a need to prove their credibility to health care providers who seemed to not believe them [25,26]. Some IBS patients felt dismissed by health care providers. Candid feedback included: “*He/she did not listen or understand*”; “*The doctor thought it is all in my head*”; “*He/she never laid a hand on me!*”; and “*He/She was too busy looking at the computer screen*”.

Patients need and expect empathy and validation in order to feel better. They consider the relationship quality of medical care as important as the provider’s knowledge. In an expressive writing study, 70% of patients who were asked to write about their experience with IBS wrote about their relationship with health care providers (HCP), indicating that this relationship is an important part of patients’ illness experience. Approximately half (54%) of the relationship comments were judged to be “negative”, 11% were “positive”, and 35% were “neutral”. The most common themes were: “I need more empathy and my HCP needs to understand how much IBS affects my life” (27%); “Nothing my HCP does helps my IBS” (25%); “My HCP has been helpful and reassuring” (17%); “My HCP thinks I’m crazy” (8%); and “I don’t trust my HCP” (5%). Overall, the patients considered that the most important components of building the relationship are listening, empathy, and receiving education about IBS. Contrary to common beliefs, in the same study, only 4% endorsed the care providers’ need to “spend more time with me”. The patients’ wish list included: “*Believe my symptoms*”; “*Listen, understand and take me seriously”; “Not tell me to “live with it*”; “*Verbally express hope*”; “*Understand how much IBS affects my daily life*”; and “*Reassure me not to give up*” [27].

Many IBS patients feel insufficiently informed about their disease and hold misconceptions that can potentially increase anxiety or drive demands for further testing and increased visits. Common misconceptions include: IBS is colitis or can result in colitis, malnutrition, and cancer; IBS can shorten a person’s life; and IBS develops because of deficient digestive enzymes or the presence of stress/anxiety or depression [28,29].

It is not uncommon for IBS patients to doubt the “functional” diagnosis. In one study, one out of 13 patients seen by a specialist accepted the diagnosis [30]. Such mistrust of the diagnosis could be related to how the diagnosis was explained. Nevertheless, this mistrust likely limits satisfaction and adherence to the treatment plan, and promotes disease-related anxiety, health care utilization, and provider-switching. Overall, the most commonly unmet patient expectations are for providers to show more empathy, to create rapport, and to provide disease-related education.

## 2. Providers’ Views on IBS

There is a significant disconnect between the views of patients and providers regarding etiology, treatment approaches, and the efficacy of treatment options for IBS [31,32]. 

When compared to their patients, providers generally: (a) underestimate the severity of IBS and the negative effects IBS symptoms have on their quality of life; (b) consider the symptoms less serious or important; (c) perceive the role of psychological contribution as a cause of IBS to a greater degree than patients do; and (d) believe patients’ requests to be less reasonable. These differences naturally lead to greater patient dissatisfaction. These differences are amplified by additional factors. Several studies suggest that more than 50% of primary care providers view IBS as a diagnosis of exclusion, and many limit treatments to fiber and antispasmotics [33,34,35]. Only about 50% of patients with IBS are provided with a definitive diagnosis after seeing a physician [36]. The uncertainty about the diagnosis, including the uncertain diagnostic language used to describe the condition to the patients, may contribute to patients’ disregard of their diagnoses and may result in increased disease-associated anxiety or more unnecessary testing and surgeries [37,38]. 

The prejudice towards IBS is part of the medical profession’s general bias about organic versus “functional” diagnoses. A study interviewing physicians reported that physicians may have a ‘public’ and ‘private’ (more prejudicial) definition of IBS. Gastroenterologists tend to be more judgmental towards IBS patients than primary care providers. Most providers admit to feeling frustrated with an uncertain diagnosis and lack of cure for IBS, as well as being intolerant of what they perceive to be a “typical” IBS patient. Some providers even make a distinction between ‘good’ and ‘bad’ IBS patients [39]. A study examining attitudes of Gastroenterology Fellows responding to patients’ after-hours telephone calls, demonstrated double standards. Gastroenterology fellows considered the calls made by patients with functional GI disorders as less serious and less reasonable, and in general symptoms to be less disabling than those for patients calling with “organic” complaints. The fellows also liked patients with an organic disease better than patients with a functional diagnosis [40]. 

The knowledge and attitudes of providers regarding IBS may vary based on their specialty, although most providers feel that IBS patients need more time per visit than other “sicker” patients. A study comparing family practitioners (FP), internists (IM), and GI physicians (GI) concluded that more gastroenterologists considered prior infection and a history of abuse as factors related to IBS, while more family practitioners believed that diet may be the cause of IBS. GI physicians estimated making a new diagnosis of IBS without the need for testing in about 42% of cases. Doctors in primary care estimated referring about 30% of IBS patients to a GI specialist. Obviously, these differences are likely related to differences in training and the ability to perform specialized testing [34]. Gender stereotyping about IBS is also prevalent in medical encounters, with women overall being at risk for being belittled and men at risk of being unnoticed due to the ‘female health concern’ tag attached to IBS [41]. 

## 3. Optimizing the Patient—Provider Interaction 

### Patient—Provider Relationship is Essential for Good Patient Outcomes

The greatest gap between IBS patients’ health care experience and their expectations relates to their interaction with providers. Patients need more empathy, in addition to more information that they can understand and apply in their daily lives. Patient dissatisfaction relates to both the content of the information provided and how this is presented [40]. 

A patient-centered approach (one that revolves around the patient and emphasizes self management) with a strong focus on improved communication is increasingly recognized to be essential when managing patients with IBS or chronic illnesses in general [42,43]. 

In attempts to develop evidence-based guides for teaching and learning communication skills, several consensus statements for P-P communication have been developed, such as Calgary Cambridge Referenced Observation Guides, the UK Consensus Statement, the Kalamazoo Consensus Statement, and the German Basel Consensus Statement [44,45,46,47]. 

The essential elements of communication in medical encounters can be summarized in seven essential sets of skills: (1) build the provider-patient relationship; (2) open the discussion; (3) gather information; (4) understand the patient’s perspective; (5) share information; (6) reach agreement on problems and plans; and (7) provide closure [47]. Examples of how these elements can relate to P-P interaction in IBS management are provided in Table 1. 

Communication skills can be learned and practiced, leading to significant positive effects on patient satisfaction and health outcomes, as well as to increased provider job satisfaction [48,49]. Generally, providers need more training concerning the diagnosis and treatment of IBS, and recognizing and treating psychiatric co-morbidities. Improvements in these areas will nurture an environment for an improved therapeutic P-P relationship, optimized quality of patient care, and better clinical outcomes.

## 4. Patient Education in IBS

Knowledge is empowering. As with most chronic diseases where there is no cure, the role of the provider is to help the patients to self-manage their symptoms and to help them feel in control of their illness. Providing education tailored to the patient’s level of informational need is therefore a very important part of managing IBS. In addition, as previously discussed, providing disease-specific knowledge is an important relational aspect of patient care. 

Knowledge is thought to be stored in the form of “conceptions”, which are coherent identifiable ideas that combine factual knowledge, values, and beliefs. Learning occurs when existing conceptions are modified or exchanged for new and more convincing ones. Unless prior preconceptions are identified, learning may be blocked because the existing conceptions affect what and how people learn. Therefore, effective education starts by eliciting a patient’s prior knowledge and educational needs before introducing new ideas and concepts.

Misconceptions are widespread, particularly relating to the causes and prognosis of IBS. Many IBS patients believe that diet alone or anxiety and depression can cause IBS. Significant numbers of IBS patients believe that IBS can lead to cancer, colitis, or malnutrition, shorten their life, or require surgery. Correcting these misconceptions reduces disease-related anxiety. Topics of patients’ interest include dietary modifications, the cause of IBS, coping strategies to reduce symptoms, and how to prevent IBS attacks [42,50,51]. 

Several IBS educational approaches have been studied ranging from self-help interventions such as guide books [52,53,54], cognitive behavior-based self-management [55], and one vs. multiple sessions of IBS education, alone or as apart of multicomponent intervention [55,56,57]. It appears that even a brief one-time psycho-educational group intervention in the form of an IBS class, consisting of information on diet, a healthy lifestyle, and general information regarding pathophysiology and IBS symptoms, is efficacious in changing cognitions and fears about IBS and in improving disease-related quality of life. 

Group education appears to be particularly well-suited for IBS because of its lower cost and the added benefit of allowing for sharing of experiences and patient-to-patient peer support [56,57,58]. 

For patients with severe IBS who have significant psychological and psychiatric co-morbidities, education alone would probably be ineffective, and behavioral interventions would be more beneficial. Fortunately, these patients represent only a relatively small proportion of IBS health care seekers. For the majority of IBS patients with mild/moderate IBS, using an individual or group educational intervention tailored to the educational needs of the patients may provide the proper level of educational intervention to address their needs and goals at a lower cost. Furthermore, the approach is more feasible than genuine multicomponent behavior therapies requiring a trained mental health professional.

Patient education is most effective when it is individualized to a patient’s specific needs. Therefore, it is not possible to design a script that will be helpful for all IBS patients. However, there are a few key educational points that will benefit most IBS patients, as presented in Table 2. 

IBS is a poorly understood and highly prevalent disorder with significant negative effects on patients’ quality of life. Providers and patients share a common goal towards the best care possible but often differ in their views regarding IBS, which may lead to an impaired P-P relationship and diminished clinical outcomes. Patients need to work on the self-management of IBS symptoms in partnership with their health care providers. Providers need to increase their knowledge regarding IBS, better appreciate the effects of this common disorder on patients’ lives, and provide greater empathy, education, support, and hope. Ultimately, providers need to appreciate that the relationship aspects of care are therapeutic and as important for clinical outcomes as the pharmacological treatments they prescribe. A patient-centered approach with an emphasis on effective communication is essential when helping patients manage IBS and when dealing with illnesses in general.

## Figures and Tables

**Table 1:** Elements of Patient—Provider communication.

Elements of Communication	How to	Example
**Build the relationship**	Greet warmly	Use eye contact and smile when greeting the patient
	Elicit full agenda set priorities early in the interview	“*Is there anything else bothering you?*”
		“*How can I help you the most today?*”
**Listen actively**	Start with open ended questions	“*Tell me more about the pain*”
	Use silence—repress the desire to respond with advice or an opinion (do not interrupt particularly in the first few minutes)	Nonverbal elements of active listening (directly face the person with open body relaxed posture, maintain eye contact, lean forward, head nodding)
	Paraphrasing—rewarding a statement usually with less words	“*You had severe pain for a very long time*”
	Clarifying—transforming unclear information into clear	“*Sounds like you have seen many doctors and tried quite a few treatments without much success*”
**Use Empathy** The ability to understand the feelings of another person	Encourage emotional expression	“*How does this situation make you feel?*”
	Identify and accept/validate feelings	“*I can see how difficult it has been for you to cope with these severe symptoms*”
	Demonstrate empathy—verbally and non verbally	“*It must be very frustrating to feel that no one understands.*”
**Elicit patient’s perspective**	Patient’s beliefs regarding the illness	“*What do you think is the cause of your illness?*”
		“*What do your family and friends think about your condition?*”
	What is the impact on the quality of life	“*How are these symptoms affecting your life?*”
	Disease-related worries/anxiety	“*What are you worried about in relation to your IBS?*”
**Provide education**	Elicit prior knowledge and educational needs	“*What would you like to know about IBS the most?*”
	Correct misconceptions	“*IBS does not become cancer or colitis*”
	Facilitate learning through problem solving	“*Let’s think what can you do if you are at work when the pain starts?*”
	Test for comprehension	“*Can you tell me what you understood about this medication so far?*”
**Negotiate a mutual treatment plan**	Use patient’s frame of reference	“*You described the burning sensation that …*”
	Involve the patient in the decisions	“*Which of the treatments we talked about are you most interested in trying?*”
		“*What do you think will help the most?*”
	Explore plan acceptability/barriers	“*Do you think you will be able to stick to this plan?”*
		“*How can we make it easier?*”
	Set a realistic goals	“*Lets agree on working on making the symptoms better even though we may not be possible to make them go away completely*”
	Encourage questions	“*What questions do you have about..?*”

**Table 2:** Key educational points that benefit most IBS patients.

Topic	Importance
IBS is a real gastrointestinal condition (not “in your head”).	*Provides validation and demonstrates empathy*
IBS can significantly affect one’s life.	
IBS is a chronic medical condition, although the symptoms can come and go. There is no magic pill for IBS.	*Helps patients set realistic expectations*
There are many things we can do to help you better manage IBS symptoms.	*Provides hope to the patient, while implying the need for self management*
You may have long periods of time (sometimes years) without experiencing any symptoms.	
IBS does not cause cancer, colitis, or any other problems. It does not shorten your life.	*Helps to clarify potential common misconceptions and reduce disease-related anxiety*
For some people with IBS, stress can trigger symptoms or make them worse.	*Can be used to further explore the role of psychological factors in IBS*
We need to work together to help you manage your IBS.	*Emphasizes the need for a collaborative approach*
